# Surgical Management of Paediatric Aphakia in the Absence of Sufficient Capsular Support

**DOI:** 10.1155/2021/2253486

**Published:** 2021-12-04

**Authors:** Evdoxia-Maria Karasavvidou, Craig Wilde, Anwar Zaman, Gavin Orr, Dharmalingam Kumudhan, Georgios D. Panos

**Affiliations:** ^1^Karasavvidou Eye Centre, Naousa, Greece; ^2^Department of Ophthalmology, Queen's Medical Centre, Nottingham University Hospitals NHS Trust, Nottingham, UK

## Abstract

There are several available options for the demanding surgical correction of paediatric aphakia without sufficient capsular support. The literature suggests the implantation of a transscleral fixated posterior chamber-intraocular lens (PCIOL), an intrascleral fixated PCIOL, an iris-sutured intraocular lens (IOL), or an anterior chamber iris-claw IOL. We searched for reports on the management of paediatric aphakia in case of inadequate capsular support that delineated the diverse surgical approaches and their postoperative results. Analysis demonstrated that different complications can be encountered depending on IOL placement technique, such as suture rupture, IOL dislocation, secondary glaucoma, endophthalmitis, vitreous hemorrhage, and endothelial cell loss. However, it was shown that various IOL designs have similar visual outcomes. Taking into consideration the advantages and disadvantages of each surgical technique, ophthalmic surgeons can determine the safest and most efficient approach for paediatric aphakic patients.

## 1. Introduction

Paediatric aphakia with the absence of adequate capsular support may occur after lens removal for congenital cataract, after trauma or lens subluxation associated with systemic disorders. Refractive error can be temporarily managed with spectacles or contact lenses; however, secondary implantation of an intraocular lens (IOL) provides better visual outcomes in children with aphakia or lens subluxation [1, 2].

Various alternatives have been proposed for IOL secondary implantation through the years, but the research for the optimal surgical approach and IOL design is still in progress. To date, available options include the use of an anterior chamber IOL, a scleral fixated (SF) sutured posterior chamber IOL (PCIOL), an intrascleral fixated PCIOL, an iris-sutured PCIOL, or iris-claw lens [3]. Glued sutureless intrascleral fixation techniques as well as the creation of scleral pockets for intrascleral fixation of IOL haptics have shown good results in adults [4, 5]. On the other hand, the implantation of anterior chamber IOLs has been related to several postoperative complications, such as corneal endothelial cell loss (ECL), glaucoma, intraocular inflammation, hyphema, and cystoid macular oedema. Thus, anterior chamber IOLs are not recommended for use in the paediatric population [6].

This review aims to present the different surgical methods used in cases of paediatric aphakia without adequate capsular support and give a full description of their complications and visual outcomes.

## 2. Transscleral Fixation with Sutures

Over the past years, transscleral-sutured fixation of a single- or three-piece IOL has been considered an effective approach in the management of aphakia in paediatric eyes with no capsular support [7, 8]. Nevertheless, various studies have reported significant late complications related to the sutures. These complications involve suture erosion and dislocation of the IOL due to suture breakage [2, 9]. Moreover, ocular inflammation and discomfort may develop in the eyes with protruding suture ends. Close observation is required in these cases, as the risk for delayed onset endophthalmitis is increased [10, 11].

Asadi et al. showed a relatively high percentage of IOL dislocation resulting from suture rupture, a complication that was recorded in six of twenty-five paediatric eyes that underwent transscleral PCIOL [12]. Another study that evaluated the long-term outcomes of this surgical technique in children found that suture breakage was present approximately five years after surgery. They also observed that 10-0 polypropylene sutures had been used in all the cases complicated by suture rupture and that this rupture was mostly spontaneous [7]. Their results were supported by Price et al., who found degradation of the ruptured 10-0 polypropylene sutures after microscopic examination in patients with late dislocation of scleral-sutured IOLs [13]. It appears that long-term reliability of transscleral IOL fixation depends on the size and sturdiness of the suture material. Therefore, alternative suture size and materials such as 9-0 polypropylene or Gore-Tex have been suggested, considering the major concerns about the stability and safety of 10-0 polypropylene sutures used for IOL scleral fixation [7]. It is presumed that the 9-0 polypropylene suture has higher tensile strength and can better resist biodegradation and trauma [13]. Vasavada et al. observed that the 9-0 polypropylene sutures were broken clinically; however, histopathological analysis of the broken sutures was not indicative of any degenerative changes [14]. Apart from biodegradation, a clinicopathologic study concluded that polypropylene suture breakage and subsequent transscleral fixated IOL subluxation may result from the positioning IOL holes cutting the sutures and chronic inflammation that accelerates degradation process [15].

The use of alternate suture materials for IOL fixation to the paediatric sclera, including 10-0 mersilene and CV-8 Gore-Tex, has been investigated in a few studies. Although results have been encouraging, longer follow-up periods are required in order to reach safe conclusions about the stability of these materials [2].

Taking into consideration the results from published studies that investigated the use of scleral-sutured PCIOLs in children, satisfying visual outcomes have been demonstrated, with the mean postoperative best corrected visual acuity (BCVA) ranging from 0.69 ± 0.69 to 0.12 ± 0.13 logMAR at follow-up from 3 to 200 months (Table 1).

## 3. Intrascleral Fixation without Sutures

Long term side effects related to sutures led to the development of novel surgical methods for scleral IOL fixation in children. Kumar et al. presented a sutureless glued intrascleral single- or three-piece IOL fixation in 41 eyes [16]. The technique they used included externalisation of the haptics through 20G sclerotomies under partial thickness scleral flaps, which were then closed with fibrin glue. One case of postoperative optic capture and two cases of IOL decentration were reported. Similar results were demonstrated by Kannan and colleagues, who described a method of intrascleral IOL fixation without flaps, sutures, or glue in 40 eyes of 25 children with ectopia lentis [17]. The haptics were externalised through a scleral tunnel with the use of a 24G needle and buried in an adjacent scleral pocket. Four eyes developed early hyphema, five eyes developed intraocular haemorrhage, and there was one case of hypotony and one case of late IOL subluxation. Shuaib et al. compared sutureless transscleral technique for IOL fixation to retropupillary iris-claw IOLs in 30 paediatric eyes with aphakia [18]. For the IOL fixation to the sclera, they exteriorised the haptics through 23G sclerotomies under a scleral flap without glue usage. Postoperatively, hypotony due to subconjunctival leakage (*n* = 1), high intraocular pressure (*n* = 2), subconjunctival haptic exposure without erosion (*n* = 3), and IOL dislocation (*n* = 2) were mainly observed in the sutureless transscleral IOL fixation group. In a case series presented by Sternfeld et al., the flanged intrascleral IOL fixation (known as Yamane technique) was performed in order to correct aphakia in 12 eyes of 10 children [3]. In this technique adapted for paediatric patients, the haptics were externalised using a 30G thin-walled needle. Afterwards, the end of each haptic was broadened into a flange with low-temperature cautery and haptics were depressed back into the intrascleral tunnel. There was one case of postoperative IOL subluxation that also developed mild hypotony and choroidal effusion with no clinical leakage. Mild IOL decentration (*n* = 2), pigmentary deposits on the IOL (*n* = 3), irregular peaked pupil due to a vitreous strand (*n* = 1) and visible haptic through the conjunctiva in a child with Marfan syndrome were reported. Finally, a recent study retrospectively evaluated the use of Carlevale IOL in five paediatric eyes with aphakia and insufficient capsular support [19]. The Carlevale IOL is a novel foldable, acrylic, one-piece lens with T-shaped haptics, specifically designed for scleral fixation without sutures [20]. In the technique presented by Gotzaridis et al., the Carlevale IOL was inserted into the anterior chamber after three-port pars-plana vitrectomy [19]. The leading T-shaped haptic was then grabbed with 25G intraocular forceps under the scleral flaps and expressed through the sclerotomy. Identically, the trailing haptic was expressed through the other sclerotomy. No significant complications were reported after the surgery, and in all cases, IOL was well centered without IOL capture.

Visual outcomes of intrascleral fixated PCIOLs without sutures in paediatric population are comparable to those that have been reported for scleral-sutured IOLs. Kumar et al. found that the mean postoperative BCVA for glued intrascleral fixated PCIOLs was 0.43 ± 0.33 at a follow-up period of 17.5 ± 8.5 months (range 12–36 months) [16]. They also noted that in 53.6% of the cases, BCVA improved by more than 1 line, with no BCVA loss in any other case. Similar BCVA improvement was reported in 47.5% of the eyes that had intrascleral fixation of IOL haptics with no sutures or glue [17]. Visual results of the flanged intrascleral IOL fixation (Yamane technique) agree with those presented above, since postoperative BCVA improved in 50% of the cases or remained stable. Significant visual acuity improvement has been reported with Carlevale IOLs as well, with a mean postoperative BCVA at 0.26 ± 0.32 logMAR after a median follow-up period of 9 months (range 7–13 months) [19]. Although all these sutureless methods appear promising, they have not been widely performed in children and their long-term results need to be investigated.

## 4. Iris-Sutured IOLs

The use of iris-sutured IOLs has also been proposed for the correction of aphakia in children with no adequate capsular support. Dureau et al. described a surgical method for iris fixation of foldable IOLs in 17 eyes of 9 paediatric patients with ectopia lentis [21]. Postoperatively, they found one case of hyphema and one case of aseptic endophthalmitis; however, in all cases, IOLs were centered and pupils were round [21]. Another study presented the outcomes of iris-sutured IOL implantation in 12 eyes of children with ectopia lentis. IOL dislocation without breakage of fixation sutures was detected in four eyes (33%). They hypothesised that these dislocations resulted from the rotation of the IOL haptic out of the suture loop [22]. Although a previous report claimed that using the remaining portion of the capsule with sufficient zonular support may be helpful in achieving better IOL stability [21], Kopel and colleagues supported that capsular remnants may be the cause of vitreous traction resulting in retinal tears and/or detachment [22]. In a similar research by Yen et al., dislocation of the iris-fixated IOL was observed in 41% of the cases [23]. Nevertheless, it was highlighted that all sutures were intact and in their appropriate iris position. Finally, researchers described the rare development of a secondary iris cyst after iris-sutured IOL insertion in two children with Marfan syndrome [24, 25].

Postoperative BCVA in paediatric cases that were corrected with iris-sutured IOLs varied from 0.24 to 0.35 logMAR at the last follow-up of 19.8 months to 4.7 years [2]. In a case series that compared the outcomes of pars-plana lensectomy-vitrectomy with and without iris-sutured IOL in paediatric eyes with ectopia lentis, no statistically significant difference in the fraction of eyes that achieved BCVA of 20/40 or better was found between the two groups [22]. In addition, no difference was observed in mean postoperative BCVA. On the other hand, Shah et al. noticed that mean visual acuity improved in 71% of the cases but decreased in another 24% after iris-sutured IOL placement [26].

## 5. Anterior-Fixated Iris-Claw IOLs

Another alternative solution for the correction of paediatric aphakia in case of insufficient capsular support has been the implantation of Artisan IOL [27]. The Artisan IOL, invented by Worst in 1986, was originally a biconcave iris-enclavated IOL used in phakic patients with high myopia [28]. Due to its different design, the current biconvex rigid acrylic three-piece IOL allows aqueous flow between the optic and the iris and decreases pigment dispersion. IOL haptics are fixated to the peripheral iris, at 3 and 9 o'clock, with the use of an enclavating needle [2].

Anterior-fixated Artisan IOLs prevail over scleral- or iris-sutured IOLs because their implantation is technically easier [29]. In addition, the risk of posterior segment complications is reduced as no surgical manipulation takes place behind the iris. Nevertheless, such complications have been reported, but their occurrence is attributed to multiple factors, including concomitant surgical procedures such as vitrectomy or a history of previous surgery. Long axial lengths or other structural ocular deformities seem to be equally significant. For instance, axial elongation and higher lens subluxation probability appear to increase the risk of retinal detachment in patients with Marfan syndrome [2].

Anterior chamber Artisan IOLs may be complicated by pupillary block, with a cited frequency ranging from 0% to 20%, and this complication can be detected from day 1 to 9 months postoperatively [2]. Hirashima et al. found that pupillary block can still be observed in pseudophakic eyes, even after laser iridotomy [29]. Surgical iridectomy was successfully performed for the inversion of pupillary block in all the reported cases. Therefore, the implantation of Artisan IOL should always involve a surgical peripheral iridectomy with sufficient size, in order to avoid this complication [2].

De-enclavation of Artisan IOLs has been recorded in up to 6.25% of cases [2]. In a study by Tychsen and Faron, repeated IOL de-enclavation led to the explantation of the Artisan IOL in one of the total 28 eyes that they were implanted [30]. Manning and colleagues reported spontaneous lens de-enclavation in one of the 16 eyes with ectopia lentis that had been treated with Artisan IOL implantation [31]. This complication occurred at the seventh postoperative year. A similar condition was described in a case series by Ong et al. [32].

The preservation of endothelial cell count (ECC) is a challenging issue for ophthalmic surgeons when performing cataract surgery and lens implantation [2]. Over the years, many studies have investigated ECL after lensectomy in children. In a prospective study by Kora et al., ECL was evaluated to be 6% three years after cataract surgery, using PCIOL and Hessberg-type anterior chamber IOLs [33]. They assumed that factors, such as age, indications for surgery, or technique, could change that result. The degree of ECL varied from 5.3% to 7.5% after extracapsular cataract surgery and PCIOL implantation in another long-term study on paediatric population [34]. Ramasubramanian and colleagues showed that mean ECC was decreased by 11% in paediatric eyes that had undergone cataract surgery [35].

Artisan IOLs concern specialists even more about ECL, because they were created for anterior chamber placement. In a number of studies that investigated the long-term results of Artisan IOLs in children with lens subluxation related to systemic disorders, the mean ECL was found to be higher, ranging from 14.2% to 18.5% [2]. Nonetheless, there are some reports that display conflicting results. Güell et al. discovered that mean ECC was not statistically different in eyes with Artisan IOL compared to eyes with any IOL-type placement [36]. Two other studies on children with lens subluxation concluded that there was no statistically significant difference in ECL between unoperated eyes and eyes that had undergone lensectomy and Artisan IOL implantation [27, 37].

It appears that prior ocular trauma may lead to even lower ECC. Odenthal and colleagues retrospectively assessed ECL after the implantation of Artisan IOL for congenital and traumatic cataract in children [38]. They observed that mean ECL was 41% in the traumatic cataract group after 10.5 years of follow-up and was strongly correlated with the original corneal scar length from trauma. Similarly, in a long-term study by Gawdat et al., a higher rate of ECC loss was noted in paediatric eyes that had undergone Artisan IOL implantation for the correction of aphakia after traumatic cataract surgery at 12-month follow-up [33]. It was assumed that original trauma and its surgical repair were liable for the greater ECL rate. However, they highlighted the necessity of longer follow-up, in order to evaluate ECL through adulthood.

Various factors are presumed to affect ECL, besides trauma. Different ECL rates have been presented among studies that explored the outcomes and complications related to Artisan IOLs and this diversity probably results from the wide variation in described surgical techniques, incisions, and follow-up durations. Considering the high heterogeneity of ECL results, researchers have not reached a safe conclusion about the correlation between ECL and the use of anterior-fixated Artisan IOLs [2].

Even though anterior Artisan IOLs have been associated with serious complications, encouraging visual outcomes have been published by multiple studies over the last two decades (Table 2). Research results showed that visual acuity was stable or improved in cases of lens subluxation that were managed with Artisan IOL placement. The mean postoperative BCVA varied from 0.36 ± 0.26 to 0.04 ± 0.09 logMAR at different follow-up durations [2].

## 6. Retropupillary-Fixated Iris-Claw IOLs

An alternative, retropupillary fixation of Artisan IOL has been recommended, in order to avoid ECC issues that result from anterior-fixated Artisan IOLs. Studies on the adult population have shown that, since retropupillary-fixated Artisan IOLs are located in the posterior chamber, the depth of the anterior chamber increases. Thus, the risk for ECL, which is the main concern when Artisan IOLs are used, is hypothetically lower [39, 40]. Gonnermann and colleagues presented the outcomes of retropupillary-fixated Artisan IOL use in seven paediatric eyes with lens subluxation and no capsular support [41]. A mean ECL of 6.4% was noted at the last postoperative follow-up, without spontaneous IOL dislocation or any other reported serious complication. Similar studies also revealed low complication rates with this technique [42, 43].

Retropupillary fixation of Artisan IOL has demonstrated good visual outcomes in aphakic children. In the study by Gonnermann et al., the mean postoperative BCVA was 0.13 ± 0.17 logMAR at a follow-up duration of 31 ± 21 months (range 10–64 months) [41]. These results are comparable to those reported for anterior-fixated Artisan IOLs and scleral fixated IOLs.

## 7. Conclusions

Surgical correction of paediatric aphakia with inadequate capsular support represents a challenging task for ophthalmic surgeons. Several IOL designs and surgical techniques have been recommended, each one with benefits and risks. The literature has shown that scleral fixation provides good refractive and visual outcomes. Nevertheless, this method has demonstrated higher rates of complications associated with the sutures, such as suture erosion and IOL dislocation, as well as ocular inflammation and endophthalmitis. Similar complications have been described with iris-sutured IOLs, except suture breakage. Fewer cases of suture-related IOL decentration have been encountered with sutureless intrascleral IOL fixation, including the Yamane technique or the implantation of the novel Carlevale IOLs. These alternatives are potentially less complicated, but also faster. Finally, the placement of anterior Artisan IOLs is reportedly easier than that of the previous techniques; however, their long-term impact on ECL still needs to be determined.

The current literature for the correction of aphakia in children has several limitations. The majority of published reports are retrospective and noncomparative, and their sample size is small. Therefore, larger prospective studies are required in order to define which one is the optimal approach for the management of aphakia in the paediatric population.

## Figures and Tables

**Table 1:** The most important studies of scleral-sutured IOLs in paediatric population.

Study	Design	Number of patients (eyes)	Key results
Sharpe et al. (1996)	Retrospective outcomes of scleral-sutured PCIOLs	7 (7)	(1) VA improvement in six of seven patients (average improvement of 4 lines)
			(2) Complications: scleral fixation suture exposure (*n* = 1), lens decentration (*n* = 1), and lens tilt (*n* = 1)

Lam et al. (1998)	Retrospective safety and efficacy of scleral fixated IOLs	3 (6)	(1) Good visual improvement
			(2) Stable and well-positioned PCIOL after surgery in all eyes
			(3) Complications: asymptomatic pupillary IOL capture in 3 eyes

Kumar et al. (1999)	Prospective case series evaluation of scleral fixated IOL implantation	11 (11)	(1) Postoperative BCVA: stable in 54.5%, improved by more than 1 Snellen line 27.2% and decreased by more than 1 Snellen line in 18.1%
			(2) Complications: suture erosion through the conjunctiva in 18.18%, marked postoperative anterior chamber reaction in 18.18%, IOL decentration in 9.09%, glaucoma in 9.09%, and cystoid macular edema in 9.09%

Zetterström et al. (1999)	Retrospective long-term outcomes of scleral-sutured PCIOLs	13 (21)	(1) Postoperative BCVA: stable or improved
			(2) Complications: posterior synechiae (*n* = 4), cells on the IOL surface (*n* = 4), and IOL subluxation (*n* = 2); no visual axis opacification, secondary glaucoma, or retinal complication was recorded

Vadalà et al. (2000)	Retrospective results of scleral fixated IOLs	3 (5)	(1) Postoperative VA: 20/20 to 20/40
			(2) Complications: IOL dislocation (*n* = 1) and posterior capsular opacification (*n* = 3)

Jacobi et al. (2002)	Prospective evaluation of transscleral fixated IOLs	26 (26)	(1) Postoperatively, BCVA within one Snellen line was achieved by more than 80% of the patients
			(2) Complications: IOP increase in 11.5%, marked anterior chamber reaction in 15.4%, IOL decentration in 19.2%, and suture erosion through the conjunctiva in 7.4%

Sewelam et al. (2001)	Retrospective haptic position evaluation of transscleral fixated PC IOLs using UBM	20 (20)	IOL haptics located in the sulcus (55.0%), anterior to the sulcus (27.5%), and posterior to the sulcus (17.5%)

Ozmen et al. (2002)	Retrospective assessment of the visual outcome and complications of transscleral fixated IOLs	18 (21)	(1) Visual improvement of more than 2 Snellen lines in 9 eyes (42.8%)
			(2) Complications: the most severe were concurrent endophthalmitis and retinal detachment (*n* = 1); the most common were pupillary distortion, transient pupillary membrane, pupillary capture, and strabismus and anterior uveitis

Bardorf et al. (2004)	Retrospective long-term results of transscleral-sutured IOLs		(1) Postoperative VA: improved in 70%; in 51% improved by two lines or more; no patient suffered visual acuity loss
			(2) Complications: small hyphemas (7%), vitreous hemorrhage (5%), ocular hypertension or hypotony (5%) and iris capture of the IOL optic (5%); no retinal detachment or other retinal complications were reported

Buckley (2007)	Retrospective long-term outcomes of transscleral-sutured PCIOLs	26 (33)	(1) Postoperative VA: significantly improved (*P* < 0.001)
			(2) Complications: intraoperative and immediate postoperative minimal and not sight-threatening; IOL subluxation due to spontaneous 10-0 polypropylene suture breakage (*n* = 3) at 3.5, 8, and 9 years after surgery; 10 similar cases by a survey of paediatric ophthalmologists (mean, 5 years after surgery)

Asadi and Kheirkhah (2008)	Case series long-term results of transscleral fixated PCIOLs	23 (25)	(1) Postoperative BCVA: improved in 48% by >1 Snellen line; the main cause of reduced vision was corneal and retinal pathologies and amblyopia
			(2) Complications: transient intraocular hemorrhage (52%), transient choroidal effusion (8%), late endophthalmitis (4%), retinal detachment (4%), and late IOL dislocation due to breakage of polypropylene sutures after 7 to 10 years (24%)

Olsen and Pribila (2011)	Retrospective sulcus fixated, sutured PCIOL using endoscopic guidance during PPV	20 (21)	(1) Most patients had visual function improvement
			(2) Complications: suture breakage (*n* = 2) due to repeat trauma
			(3) Advantages: excellent visualization and haptic localization, optimal lens centration, buried knots, broad scleral imbrication, and minimal vitreous- and hemorrhage-related complications
			(4) Disadvantages: learning curve, increased operative time, long-term suture stability issues, and limited availability of intraocular endoscopes

Burcu et al. (2014)	Retrospective evaluation of the outcomes of scleral fixated PCIOLs	14 (24)	Median postoperative BCVA: 0.2 (min: hand motion; max: 0.8) in decimal notation (*P*=0.017); BCVA improved at least one Snellen line or remained unchanged in all eyes

PCIOL: posterior chamber-intraocular lens, VA: visual acuity, BCVA: best corrected visual acuity, IOP: intraocular pressure, UBM: ultrasound biomicroscopy, and PPV: pars-plana vitrectomy.

**Table 2:** The most important studies of Artisan IOL in paediatric aphakic patients.

Study	Design	Number of patients (eyes)	Key results
Lifshitz et al. (2004)	Retrospective Artisan IOL for idiopathic subluxated lenses	3 (4)	(1) Postoperative BCVA 6/12 or better in 3 cases that could be recorded
			(2) VA improved by 2 or more Snellen lines in all eyes
			(3) Complications: none

Odenthal et al. (2006)	Retrospective ECL evaluation after Artisan IOL for traumatic and congenital cataract	10 (10)	(1) ECL: 41% in the traumatic cataract group
			(2) ECL related to the original corneal scar length of the trauma
			(3) No statistical difference in ECC between operated and unoperated eye in the congenital cataract group

Sminia et al. (2007)	Retrospective Artisan IOL for aphakia after trauma	5 (5)	(1) Postoperative BCVA 20/40 or better in 4 eyes
			(2) Mean ECL: 40%
			(3) Complications: retinal detachment 19 months after primary injury in one eye

Hirashima et al. (2010)	Randomized controlled trial anterior vs. posterior chamber iris-claw IOL lens subluxation in Marfan	16 (31)	(1) Postoperative BCVA did not differ significantly between groups
			(2) Complications: IOL dislocation (*n* = 3) in the PCIOL group, retinal detachment (*n* = 3) in both groups
			(3) Mean postoperative foveal thickness decreased in 54.16% of the patients

Sminia et al. (2011)	Retrospective ECL evaluation after Artisan IOL for traumatic and congenital cataract	10 (20)	Postoperative mean ECC comparable to the mean normal ECC for this age group reported in the literature

Sminia et al. (2012)	Retrospective Artisan IOL for ectopia lentis in Marfan	2 (4)	Good postoperative visual outcome, no serious IOL-related complications, and ECC within the expected range for normal eyes

Cleary et al. (2012)	Retrospective Artisan iris-claw IOL for ectopia lentis	5 (8)	(1) Postoperative mean VA: 0.04 ± 0.09 logMAR (*P*=0.04)
			(2) Mean postoperative ECL: 14.2% (*P* < 0.001)
			(3) Complications: none

Siddiqui et al. (2013) 23316948	Prospective evaluation of visual outcomes and ECC after Artisan IOL for lens subluxation	11 (18)	(1) Mean postoperative BCVA: 0.26 ± 0.13 logMAR (*P*=0.001)
			(2) Mean postoperative ECL: 17.1%

Tychsen and Faron (2013)	Prospective outcomes of Artisan IOL for aphakia	17 (28)	(1) Postoperatively, BCVA improved an average 2 Snellen lines (0.18 logMAR)
			(2) Complications: pupillary block (*n* = 4) and de-enclavation (*n* = 1)

Gawdat et al. (2015)	Prospective outcomes of Artisan IOL for aphakia	18 (25)	(1) Postoperative BCVA for traumatic aphakia and lens subluxation improved to 0.38 ± 0.15 logMAR (*P* < 0.002) and 0.3 ± 0.2 logMAR (*P* < 0.0001), respectively
			(2) One year postoperative significant decrease of ECC (2892.64 ± 441.79 cells/mm^2^)
			(3) Complications: traumatic dislocation (*n* = 2), pupillary block (*n* = 1)

Manning et al. (2016)	Retrospective outcomes after Artisan IOL for ectopia lentis in Marfan	8 (16)	(1) Mean postoperative BCVA: 0.12 ± 0.19 logMAR
			(2) Mean postoperative ECL: 15.4%
			(3) Complications: pupillary block (*n* = 1) and de-enclavation (*n* = 1)

Kavitha et al. (2016)	Retrospective outcomes of Artisan IOL vs. PCIOL for traumatic cataract	50 (50)	(1) BCVA improvement in both groups with no significant difference in BCVA logMAR between them
			(2) Complications in the Artisan IOL group: secondary glaucoma (*n* = 1), IOL de-enclavation (*n* = 1), and cystoid macular edema (*n* = 1)

Catala-Mora et al. (2017)	Prospective outcomes of Artisan IOL for ectopia lentis	12 (21)	(1) Mean BCVA improved from 0.91 ± 0.29 logMAR to 0.18 ± 0.23 logMAR at final follow-up (*P* < 0.0001)
			(2) Postoperative ECL: 5.04% ± 9.58% with an annual ECL rate of 3.16% ± 4.46%
			(3) Complications: traumatic IOL dislocation and retinal detachment (*n* = 1), cystoid macular oedema (*n* = 1)

IOL: intraocular lens, BCVA: best corrected visual acuity, VA: visual acuity, ECL: endothelial cell loss, and ECC: endothelial cell count.

## Data Availability

No data were used to support this study.
